# Identifying determinants for falls among Iranian older adults: insights from the Bushehr Elderly Health Program

**DOI:** 10.1186/s12877-024-05180-1

**Published:** 2024-07-09

**Authors:** Kazem Khalagi, Amir Human Hoveidaei, Hani AziziKia, Amirali Karimi, Reza Sattarpour, Noushin Fahimfar, Mahnaz Sanjari, Mohammad Javad Mansourzadeh, Iraj Nabipour, Bagher Larijani, Afshin Ostovar

**Affiliations:** 1 Osteoporosis Research Center, Endocrinology and Metabolism Clinical Sciences Institute , Tehran University of Medical Sciences, No.10- Jalal-e-ale-ahmad st, Chamran hwy, 14117-13137, Tehran, Iran; 2 Obesity and Eating Habits Research Center, Endocrinology and Metabolism Clinical Sciences Institute, Tehran University of Medical Sciences, Tehran, Iran; 3 Sports Medicine Research Center, Tehran University of Medical Sciences, Tehran, Iran; 4 Student Research Committee, School of Medicine, Shahroud University of Medical Sciences, Shahroud, Iran; 5 School of Medicine, Tehran University of Medical Sciences, Tehran, Iran; 6 Department of Epidemiology and Biostatistics, School of Public Health, Tehran University of Medical Sciences, Tehran, Iran; 7 The Persian Gulf Marine Biotechnology Research Center, The Persian Gulf Biomedical Sciences Research Institute, Bushehr University of Medical Sciences, Bushehr, Iran; 8 Endocrinology Research Center, Endocrinology and Metabolism Clinical Sciences Institute, Tehran University of Medical Sciences, Tehran, Iran

**Keywords:** Falls, Accidental falls, Risk factors, Older adults, Iran

## Abstract

**Background:**

Falls are a common cause of fractures in older adults. This study aimed to investigate the factors associated with spontaneous falls among people aged ≥ 60 years in southern Iran.

**Methods:**

The baseline data of 2,426 samples from the second stage of the first phase of a prospective cohort, the Bushehr Elderly Health (BEH) program, were included in the analysis. A history of spontaneous falls in the year before recruitment was measured by self-report using a standardized questionnaire. Demographic characteristics, as well as a history of osteoarthritis, rheumatoid arthritis, low back pain, Alzheimer’s disease, epilepsy, depression, and cancer, were measured using standardized questionnaires. A tandem gait (heel-to-toe) exam, as well as laboratory tests, were performed under standard conditions. A multiple logistic regression model was used in the analysis and fitted backwardly using the Hosmer and Lemeshow approach.

**Results:**

The mean (standard deviation) age of the participants was 69.34 (6.4) years, and 51.9% of the participants were women. A total of 260 (10.7%, 95% CI (9.5–12.0)%) participants reported a spontaneous fall in the year before recruitment. Adjusted for potential confounders, epilepsy (OR = 4.31), cancer (OR = 2.73), depression (OR = 1.81), low back pain (OR = 1.79), and osteoarthritis (OR = 1.49) increased the risk of falls in older adults, while the ability to stand ≥ 10 s in the tandem gait exam (OR = 0.49), being male (OR = 0.60), engaging in physical activity (OR = 0.69), and having high serum triglyceride levels (OR = 0.72) reduced the risk of falls.

**Conclusion:**

The presence of underlying diseases, combined with other risk factors, is significantly associated with an increased risk of falls among older adults. Given the relatively high prevalence of falls in this population, it is crucial to pay special attention to identifying and addressing these risk factors.

**Supplementary Information:**

The online version contains supplementary material available at 10.1186/s12877-024-05180-1.

## Introduction

Falling is an unexpected event in which a person moves downward to make contact with the ground, floor, or a lower level [1]. According to the World Health Organization, falls cause 684,000 deaths yearly, making it the second leading cause of unintentional injury deaths [2]. Globally, 30–40% of people over the age of 65 fall at least once a year, with 15% of them falling twice or more [3]. For those over the age of 80, the rate is 50% [4]. A recent study showed that this number is higher in the Middle East region (Gulf Cooperation Council countries: 46.9%) [5]. About 15% of falls result in non-fatal injuries such as bruises, lacerations, and hip fractures, while 23–40% of injury-related deaths in older people are caused by falls [6, 7]. Additionally, 5–10% of falls lead to severe injuries like brain damage [8]. These non-fatal injuries can result in a loss of independence and psychological stress, increasing morbidity in older adults [9, 10]. Studies have shown that after experiencing a fall, the fear of falling again leads to decreased daily mobility, which in turn increases the risk of falling [11, 12]. Approximately 4.1% of falls result in fractures, with hip fractures being associated with the greatest decrease in mobility (a 76% decline) [13, 14]. Overall, falls account for 0.85–1.5% of all healthcare costs [15].

Falling risk factors vary worldwide. These risk factors depend on aging patterns (genetic factors that contribute to different aging conditions) as well as environmental factors such as elder safety programs. Previous studies have identified risk factors such as demographic variables, systemic conditions, cardiovascular disease, neurological and mental disorders, medication, and location [3, 16].

According to previous studies, population aging in Asia, especially in East Asia, is on the rise [17, 18]. Given the knowledge about injuries related to falls, it is crucial to determine their prevalence in different groups and the associated factors in all regions of the world. Few studies on falls among older adults have been conducted in our region, and these studies have shown relatively significant differences in results [5]. The importance of investigating these differences is to establish preventive measures. In this study, we aim to investigate falls and their associated factors in a southern province of Iran for the first time.

## Methods

### Study design

The present study is a cross-sectional analysis that utilizes the baseline data collected during the second stage of the initial phase of the ongoing Bushehr Elderly Health Program (BEHP) [19, 20]. BEHP is a multi-phasic, population-based prospective cohort study conducted in a southern city of Iran. Its focus is on studying non-communicable diseases and their associated risk factors. In the first stage of the first phase, 3000 participants over 60 years old were studied regarding cardiovascular risk factors and events [19]. The second stage of the first phase of BEHP focused on musculoskeletal and cognitive diseases and started in 2015 with 2426 participants from the previous stage [20]. Sampling for this study was done using a multistage cluster random method from the population living in 75 strata in Bushehr city. The detailed methodology of BEHP is mentioned in its protocol [19, 20]. The study was approved by the Research Ethics Committee of Bushehr University of Medical Sciences (Ethical Code: B-91-14-2) and the Ethical Board Committee of the Endocrinology & Metabolism Research Institute of Tehran University of Medical Sciences (Ethical Code: IR.TUMS.EMRI.REC.1394.0036). Enrolled patients signed a written consent form after the study’s objectives were explained by the interviewer.

### Measurements

In the second stage of the first phase of the BEHP, the data were collected through standardized questionnaires, physical examination, dual x-ray absorptiometry, and laboratory tests [20].

A history of spontaneous falls in the year before recruitment was measured through self-report using a standardized questionnaire (see Additional file 1). Demographic characteristics, as well as the history of smoking alongside past medical history including osteoarthritis, rheumatoid arthritis, low back pain, Alzheimer’s disease, epilepsy, depression, and cancer, were measured using standardized questionnaires (see Additional file 1).

The physical activity level over a 24-hour period, including sports, work, and leisure time on an average weekday, was assessed using a validated self-report questionnaire that ranked activities based on nine metabolic equivalents (0.9 to > 6 METs) [21, 22]. The time spent on each activity was multiplied by the MET level to calculate a MET.time sum for the entire day [21, 23]. All data were collected by a trained nurse. Height and weight were measured with a fixed stadiometer and a digital scale following the standard protocol. Systolic blood pressure (SBP) and diastolic blood pressure (DBP) were measured using a standardized mercury sphygmomanometer on the right arm after 15 min of rest in a seated position. The first and fifth Korotkoff sounds were recorded as SBP and DBP, respectively. The average of the two readings was calculated as the participant’s blood pressure. During the full tandem balance test, the interviewer assists by supporting one arm while participants position their feet. The interviewer then asks if they are ready and subsequently releases the support, commencing the timing. The timer is stopped either after 10 s have passed or if participants move their feet. Body composition for each participant is measured using dual x-ray absorptiometry (DXA, Discovery WI, Hologic, Bedford, Virginia, USA). This measurement includes: (1) bone mineral density in the lumbar spine and hip, (2) appendicular skeletal muscle mass, and (3) the skeletal muscle mass index. Osteoporosis was defined as a T-score ≤ -2.5 (in any sex compared to the BMD of a young, healthy Caucasian person of the same sex) at any site of total hip, spine, or neck of femur.

An overnight fasting venous blood sample of 25 cc was obtained from each participant for all laboratory tests and bio-bank studies of the first phase of the study. Laboratory tests including lipid profiles, fasting blood sugar, and HbA1c were performed under standard conditions [20].

### Statistical analysis

For describing continuous variables, we used the mean (standard deviation). For categorical variables, we presented the number (percent). The simple logistic regression model investigated the association between each risk factor and the history of spontaneous falls in the previous year. Following risk factors were assessed: age, gender, education, high levels of cholesterol, high levels of low-density lipoprotein (LDL), high levels of triglyceride, low levels of high-density lipoprotein (HDL), physical activity, osteoporosis, past medical history of disease (osteoarthritis, rheumatoid arthritis, back pain, Alzheimer, seizure, depression, and cancer), balance status, type 2 diabetes mellitus, hypertension, smoking status, and body mass index (BMI). To adjust for potential confounding effects, risk factors with a P-value of ≤ 0.25 in their association with the outcome in the previous analysis were entered into the multiple logistic model and were removed from the model based on the likelihood ratio test results. We used the strategy suggested by Hosmer and Lemeshow for model fitting [24, 25]. All analyses were carried out using STATA (Version 15.1), and a P-value of 0.05 was considered significant.

## Results

A total of 2,426 older adults participated in this stage of the BEHP, and their data were used in the analysis. The mean age of the participants was 69.34 (± 6.4) years (range: 60 to 96). Of the overall 2,426 participants, 1,166 (48.06%) were men. Table 1 shows their socio-demographic characteristics.

**Table 1:** Participants’ socio-demographic characteristics

Characteristic	No. (%)
Total	2426 (100)
Women	1260 (51.9)
**Age (years)**	
60–64	598 (24.7)
65–69	952 (39.2)
70–74	379 (15.6)
75–79	282 (11.6)
80–84	146 (6.0)
≥ 85	69 (2.8)
**Marital status**	
Single	19 (0.8)
Married	1864 (76.8)
Divorced	20 (0.8)
Widow	523 (21.6)
**Education**	
No education	800 (33.0)
Primary School	885 (36.5)
Guidance school	218 (9.0)
High school	332 (13.7)
Academic	189 (7.8)
Missing	2 (0.08)

260 (10.73%) of the participants experienced falling in the last year of the study (95% CI: 9.55–12.02). Table 2 demonstrates the association between the risk factors and falling, utilizing simple logistic model analysis, alongside the number of falls in each group. The following variables were not presented in Table 2 since the P-value of the association between fall and these variables was > 0.25: type 2 diabetes mellitus, hypertension, low levels of HDL, smoking status, and BMI. In this analysis, age 65–69 (Crude OR = 0.69, 95% CI: 0.49–0.96, *p* = 0.03), male sex (0.44, 95% CI: 0.33–0.58, *p* < 0.001), having any levels of formal education above primary school (guidance school: 0.53, 95% CI: 0.30–0.93, *p* = 0.03; high school (trended towards significance): 0.66, 95% CI: 0.42–1.02, *p* = 0.06; academic degree: 0.53, 95% CI: 0.29–0.97, *p* = 0.04), higher degrees of physical activity (0.60, 95% CI: 0.42–0.85, *p* = 0.004), and the ability to stand ≥ 10 s in the tandem gait (0.37, 95% CI: 0.27–0.50, *p* < 0.001) were significantly associated with lower falls. Meanwhile, a diagnosis of epilepsy (3.91, 95% CI: 1.47–10.37, *p* = 0.006), cancer (3.12, 95% CI: 1.30–7.50, *p* = 0.01), rheumatoid arthritis (2.86, 95% CI: 1.47–5.56, *p* = 0.002), depression (2.20, 95% CI: 1.34–3.62, *p* = 0.002), low back pain (2.17, 95% CI: 1.65–2.86, *p* < 0.001), osteoarthritis (1.83, 95% CI: 1.31–2.55, *p* < 0.001), and osteoporosis (1.57, 95% CI: 1.21–2.03, *p* = 0.001) significantly correlated with higher fall rates.

**Table 2:** Frequency of participants experienced spontaneous falling last year of the study based on the studied risk factors, and the association between the risk factors and falling using a simple logistic model

Risk factor	No. [%, (95%CI)] of participants with last year falling history	Crude OR^a^ (95%CI)	*P* value
**Age (years)**			
60–64	72 [12.04, (9.66–14.9)]	1	-
65–69	82 [8.62, (6.99–10.58)]	0.69 (0.49–0.96)	**0.03**
70–74	33 [8.73, (6.27–12.20)]	0.70 (0.45–1.08)	0.10
75–79	42 [14.89, (11.19–19.54)]	1.28 (0.85–1.93)	0.24
80–84	19 [13.01, (8.45–19.50)]	1.09 (0.64–1.88)	0.75
>85	12 [17.39, (10.14–28.18)]	1.54 (0.79–3.00)	0.21
*Sex (Male)*	80 [6.86, (5.54–8.46)]	0.44 (0.33–0.58)	**< 0.001**
**Education**			
No education	98 [12.25, (10.15–14.71)]	1	-
Primary school	106 [11.96, (9.99–14.28)]	0.97 (0.73–1.31)	0.86
Guidance school	15 [6.88, (4.18–11.10)]	0.53 (0.30–0.93)	**0.03**
High school	28 [8.43, (5.88–11.94)]	0.66 (0.42–1.02)	**0.06**
Academic	13 [6.88, (4.03–11.48)]	0.53 (0.29–0.97)	**0.04**
*High cholesterol* ^b^	97 [12.22, (10.11–14.68)]	1.25 (0.96–1.63)	0.10
*High LDL* ^c^	137 [11.74, (10.01–13.71)]	1.22 (0.95–1.58)	0.12
*High triglyceride* ^d^	72 [9.38, (7.5–11.65)]	0.81 (0.61–1.07)	0.14
*Osteoporosis* ^e^	132 [13.24, (11.27–15.48)]	1.57 (1.21–2.03)	**0.001**
*Osteoarthritis*	51 [16.72, (12.93–21.33)	1.83 (1.31–2.55)	**< 0.001**
*Rheumatoid arthritis*	12 [25, (14.77–39.05)]	2.86 (1.47–5.56)	**0.002**
*Low back pain*	179 [14.14, (12.32–16.17)]	2.17 (1.65–2.86)	**< 0.001**
*Alzheimer*	5 [20, (8.57–39.99)]	2.10 (0.78–5.65)	0.14
*Epilepsy*	6 [31.58, (14.91–54.85)]	3.91 (1.47–10.37)	**0.006**
*Depression*	21 [20.19, (13.54–29)]	2.20 (1.34–3.62)	**0.002**
*Cancer* ^*f*^	7 [26.92, (13.4–46.71)]	3.12 (1.30–7.50)	**0.01**
*Physical activity* ^*g*^	72 [9.38, (7.5–11.65)]	0.60 (0.42–0.85)	**0.004**
**Tandem gait exam**			
No standing	78 [19.5, (15.9–23.67)]	1	-
Standing < 10 s	34 [15.18, (11.04–20.49)]	0.74 (0.47–1.15)	0.18
Standing ≥ 10 s	148 [8.22, (7.03–9.58)]	0.37 (0.27–0.50)	**< 0.001**

a: Prevalence Odds Ratio

b: Total cholesterol ≥ 200 mg/dL

c: Low-Density Lipoprotein cholesterol ≥ 110 mg/dL

d: Serum triglyceride ≥ 150 mg/dL

e: Measured by Bone Mineral Density (BMD) using the dual-energy X-Ray absorptiometry method by a Hologic Discovery machine. It was defined as a T-score ≤ -2.5 (in any sex compared to the ideal BMD of a young healthy white person of the same sex) at any site (total hip, spine, or neck of femur)

f: It includes breast, bladder, kidney, prostate, colon, lymph nodes, or uterine and ovarian cancer

g: Measured using Aadahl et al. physical activity questionnaire

After adjusting for potential confounders using multiple logistic regression analysis, the overall falling rates were significantly and independently lower in men (adjusted OR = 0.60, 95% CI: 0.44–0.81, *p* = 0.001), the participants aged 65 to 74 but not the patients older than 75 (65–69 y: 0.62, 95% CI: 0.44–0.88, *p* = 0.01; 70–74 y: 0.59, 95% CI: 0.38–0.93, *p* = 0.02), higher physical activity at the time of the study (0.69, 95% CI: 0.48–0.99, *p* = 0.046), and participants with higher triglyceride levels above 150 mg/dL (0.72, 95% CI: 0.53–0.97, *p* = 0.03) (Table 3). The ability to stand more than 10 s in the tandem gait examination was also significantly associated with lower falls (0.49, 95% CI: 0.35–0.69, *p* < 0.001). Fall rates increased in people with a history of epilepsy (4.31, 95% CI: 1.54–12.07, *p* = 0.005), cancer (2.73, 95% CI: 1.09–6.84, *p* = 0.005), depression (1.81, 95% CI: 1.08–3.06, *p* = 0.02), low back pain (1.79, 95% CI: 1.33–2.40, *p* < 0.001), and osteoarthritis (1.49, 95% CI: 1.05–2.11, *p* = 0.02). As shown above, a diagnosis of epilepsy posed the highest risk of falling among the aforementioned risk factors.

**Table 3:** Independent risk factors associated with the last year spontaneous falling history in older adults after adjusting for potential confounding effects using multiple logistic regression analysis

Risk factor	Adjusted OR^a^ (95%CI)	*P* value
**Age (years)**		
60–64	1	-
65–69	0.62 (0.44–0.88)	**0.01**
70–74	0.59 (0.38–0.93)	**0.02**
75–79	1.00 (0.64–1.55)	0.99
80–84	0.84 (0.47–1.51)	0.56
>85	1.08 (0.53–2.23)	0.83
*Sex (Male)*	0.60 (0.44–0.81)	**0.001**
*High triglyceride* ^b^	0.72 (0.53–0.97)	**0.03**
*Osteoarthritis*	1.49 (1.05–2.11)	**0.02**
*Low back pain*	1.79 (1.33–2.40)	**< 0.001**
*Epilepsy*	4.31 (1.54–12.07)	**0.005**
*Depression*	1.81 (1.08–3.06)	**0.02**
*Cancer* ^*c*^	2.73 (1.09–6.84)	**0.03**
*Physical activity* ^*d*^	0.69 (0.48–0.99)	**0.046**
**Tandem gait exam**		
No standing	1	-
Standing < 10 s	0.86 (0.54–1.37)	0.54
Standing ≥ 10 s	0.49 (0.35–0.69)	**< 0.001**

a: Prevalence Odds Ratio

b: Serum triglyceride ≥ 150 mg/dL

c: It includes breast, bladder, kidney, prostate, colon, lymph nodes, or uterine and ovarian cancer

d: Measured using Aadahl et al. physical activity questionnaire

## Discussion

This study found that, adjusted for potential confounders, having epilepsy, cancer, depression, low back pain, and osteoarthritis is associated with an increased risk of spontaneous falls in older adults. Moreover, the ability to maintain a tandem gait for ≥ 10 s, being male, engaging in physical activity during the study, and having high serum triglyceride levels are associated with a decreased risk of falls.

Our results show that epilepsy can be associated with an increased risk of falling. Epilepsy, with or without seizures, can cause different fall-related injuries, including fractures (two-fold increase), concussion, and cranial hemorrhage [26–28]. One reason is directly related to the seizure episodes and loss of consciousness, while another reason is complications such as reduced bone density, dizziness/sedation, ataxia/gait disturbance (caused by hyperammonemia), and cognitive impairment caused by antiepileptic drugs [29–32]. On the other hand, low compliance with antiepileptic drugs can also lead to injuries. Antiepileptic drugs in old age have other uses as well, such as for mood disturbance and neuropathic pain [33]. Management of epilepsy in the elderly has controversial details, such as the cost-benefits of prescribing antiepileptic drugs. It seems that the best solution to decrease the risk of falling is tight medical control and consultation on avoiding risky behaviors in elders with epilepsy [28].

Based on the results of this study, it appears that one of the events that occurs in individuals with cancer is falling. More than half of diagnosed cancers occur in people over 65 years old [34, 35]. The toxic effects of chemotherapeutic treatments on different body systems may be an important cause of falls. For example, chemotherapy can cause limb neuropathy, resulting in poor balance [36]. Additionally, other complications such as muscle weakness, frailty, brain and central nervous system metastases, cognitive impairment, depression, and polypharmacy are more common in older adults with cancer [35]. Furthermore, some patients with cancer may face an increased risk of injury and fractures after falls due to factors such as osteoporosis related to parathyroid-related peptides (PTHrp), bone invasion, and metastasis [35]. Because of the nature of cancers, fall-related hospitalization can increase the mortality rate in patients. Therefore, balancing the therapeutic and toxic effects of chemotherapy, especially in high-risk patients, is recommended. Recent studies show that performing a geriatric assessment before cancer treatment could decrease its negative impacts [37].

Neurological diseases (such as Alzheimer’s disease, Parkinson’s disease, and stroke) can increase the risk of falling, and as high as 60–80% of the elderly with dementia experience falling annually. For many years, gait and postural instability were considered the major risk factors for falling; however, some studies have shown that elders’ cognitive state also plays an important role [38–40]. The exact link between depression and falls is unclear, but a recent study showed that isolation and depressive symptoms could increase the risk of falls by 30% [41]. Maintaining balance requires a fast and accurate response to perturbation. This response includes physiological and cognitive factors that all come together as reaction time [42–44]. Due to the increasing prevalence of depression, cognitive assessment must be a part of any preventive program. However, the main challenge is whether to treat depression in fall-prone elders or not. Antidepressants often have side effects such as orthostatic hypotension, impaired attention, and movement disorders. Additionally, studies have suggested polypharmacy and withdrawal syndrome as risk factors for falling [45, 46]. In this regard, other approaches like Cognitive Behavioral Therapy could be helpful [46, 47].

Musculoskeletal pains increase with aging. Back pain is more common in developing countries [48, 49]. It causes limited physical activity, reduces muscle strength, and has psychological effects such as isolation [38, 50]. Recent studies have focused on low back pain as an independent risk factor for falling [51, 52]. In a recent study, Wong et al. found that chronic lower back pain is significantly associated with osteoarthritis [53]. Patients with low back pain or osteoarthritis have extended degrees of disability, increasing the risk of falling. It also has a synergetic negative effect on the quality of life and abilities of elders [54, 55]. These two factors can also cause higher morbidity after falls, such as fractures [56]. Some studies have shown that low back pain is associated with abdominotrunkal muscle weakness, and patients with pain are predisposed to falling due to its psycho-cognitive effects [57, 58].

We suggest physical activity as a protective factor against falls among the elderly. Exercise and physical activity reduce physical and mental risk factors for falling, prevent sarcopenia, and improve balance and Body Mass Index (BMI) among underweight subjects, as past studies have shown that lower BMI increases fall risk [4]. Additionally, the elderly who exercise more often have less fear of falling as a psychological factor [59]. However, while usual physical activity is beneficial, daily activities like walking need to be a part of a comprehensive strength and balance training program to be effective, as routine life activity cannot be considered an effective tool for preventing falls [60].

We suggest a tandem gait test for assessing the risk of falling in older adults. Our results show that older adults who are able to stand for at least 10 s in the tandem gait exam are less likely to fall. Some recent studies focus on gait features as an accurate classifier for the risk of falling in older adults [61].

Our findings show that high levels of serum triglycerides could be a preventive factor for falling in older adults. The adverse effects of metabolic syndrome in middle age are not applicable to everyone. However, some findings show that in elderly people, metabolic syndrome and its related components can have a positive impact on daily and cognitive function [62]. One possible explanation is that elderly individuals with higher levels of serum triglycerides may lack other more significant risk factors such as muscle mass loss, grip strength, poor nutritional state, etc., which play a greater role in the risk of falling. Another possible explanation at the molecular level involves peroxisome proliferator-activated receptor (PPAR-gamma). PPAR-gamma plays a role in fat tissue differentiation and fatty acid metabolism. More importantly, it has an anti-inflammatory and protective role against cell apoptosis in the central nervous system and skeletal muscles [63, 64]. Further studies are needed to determine the threshold for treating high levels of serum triglycerides in individuals at high risk of falling.

Our results show that older women are more prone to falling. Despite the lower mortality rate of falls in women, conditions such as hip fractures are more common in women. One possible explanation is that the aging process in women is associated with greater muscle mass loss.

Due to the multifactorial nature of falls, we need to approach them differently and design programs that take into account the unique characteristics of each population. Studies have shown that these interventions can reduce falls by 20-40% [65]. Programs such as Stopping Elderly Accidents, Deaths & Injuries (STEADI) (designed by the CDC), the Otago Exercise Program, or the Lifestyle-integrated Functional Exercise Program focus on modifiable risk factors and include screening conducted by physicians to implement preventive measures such as medication adjustment, balance improvement, and physical therapy [60, 66, 67]. Such programs are necessary due to the increasing population of individuals over 65 years old, and especially those over 85.

Since the BEH program did not include people living in nursing homes, our findings may underestimate the rate of falls [20, 68]. Additionally, when interpreting the results, attention should be given to the cross-sectional nature of the study design and the possibility of a reverse causation phenomenon, as well as the potential presence of unknown confounding factors. Furthermore, some studies suggest that low back pain is associated with osteoarthritis, which could lead to misinterpretation if each of these factors is considered an independent risk factor for falling [51].

### Limitations and strength

This study was conducted using data from a large cohort study that focused on the health of the elderly. The aim of the study was to determine the factors contributing to spontaneous falls in this population. The large number of participants and the specific target population of this cohort made it appropriate for the purpose of this study.

However, this study does have some limitations. The scope of the study did not permit a comprehensive analysis of rare diseases or risk factors like alcohol abuse. Specific subtypes of the underlying conditions mentioned, such as various types of epilepsy or specific cancer subtypes, were not examined in detail. Moreover, the absence of data on potential risk factors for falls (e.g., number of medications) limited the ability to study the impact of these factors or address their potential confounding effects. Lastly, as the study was based on cross-sectional data from the baseline measurements of the BEHP, we can only report associations and not establish causal relationships. Further research utilizing follow-up data from cohort studies is necessary to establish causal relationships.

## Conclusions

The elderly are at an increased risk of falls and subsequent complications. The presence of underlying diseases, combined with other risk factors such as older age, lower physical activity, and female sex, is associated with a significantly increased risk of falls among older adults. Therefore, this population requires preventive measures to identify and address their increased risk of falls, as well as specific attention and care to minimize the incidence of falls and their complications.

### Electronic supplementary material

Below is the link to the electronic supplementary material.

Supplementary Material 1

## Data Availability

Data supporting this study are available from the principal investigator (Dr. Iraj Nabipour, Email: inabipour@gmail.com) of Bushehr Elderly Health (BEH) program. However, the data were used under license for the current study and are not publicly available; so, restrictions apply to the availability of these data.
