# Supplementary material for: Identifying determinants for falls among Iranian older adults: insights from the Bushehr Elderly Health Program
Source: BMC Geriatr. 2024 Jul 9;24:588. doi: 10.1186/s12877-024-05180-1 (PMC11232168; doi:10.1186/s12877-024-05180-1)
Supplement: Supplementary file 1 — Supplementary Material 1 [file 12877_2024_5180_MOESM1_ESM.pdf]

English language version of some of BEH program standardized questionnaires

---

Demographic Information

- Date of Birth: Day ... Month ... Year ...

- Gender: 1- Male … 2- Female …

- Marital Status:
  1- Married … 2- Single … 3- Separated … 4- Divorced … 5- Widowed…

Smoking History

1- Are you currently smoking cigarettes, using a hookah, or smoking a pipe?

A) Cigarettes ...

B) Hookah ...

C) Pipe ...

Response Codes:

1 = Yes, regularly (at least one cigarette, one hookah session, or one pipe session per day)

2 = Yes, occasionally

3 = No

2- Did you smoke cigarettes, use a hookah, or smoke a pipe in the past? (For those who do not currently smoke)

A) Cigarettes ...

B) Hookah ...

C) Pipe ...

Response Codes:

1 = Yes, regularly (at least one cigarette, one hookah session, or one pipe session per day)

2 = Yes, occasionally

3 = No

....

History of Falling in the Past Year

Have you had a history of falling in the past year?

1 = Yes, due to an accident

2 = Yes, spontaneously

3 = Yes, due to an accident or spontaneously

4 = No

99 = I don't know

....

Medical History

- Have you ever been told by a doctor or other healthcare staff that you have the following diseases:

A) Alzheimer's (Dementia) ...

1= Yes, 2= No, 3= I don't know

- If yes, has a treatment been prescribed by the doctor for you? ....

1= Yes, 2= No

B) Rheumatoid Arthritis ...

1= Yes, 2= No, 3= I don't know

- If yes, has a treatment been prescribed by the doctor for you? ....

1= Yes, 2= No

C) Osteoarthritis ...

1= Yes, 2= No, 3= I don't know

- If yes, has a treatment been prescribed by the doctor for you? ....

1= Yes, 2= No

D) Seizures (Epilepsy) ...

1= Yes, 2= No, 3= I don't know

- If yes, has a treatment been prescribed by the doctor for you? ...

1= Yes, 2= No

E) Depression ...

1= Yes, 2= No, 3= I don't know

- If yes, has a treatment been prescribed by the doctor for you? ....

1= Yes, 2= No

F) Cancer ...

1= Yes, 2= No, 3= I don't know

- If yes, specify the type of cancer: ……
- If yes, has a treatment been prescribed by the doctor for you? ....

1= Yes, 2= No

- ➢ Have you had a history of low back pain? ....

1= Yes, 2= No
